# The association between rapid growth and lipid profile: a systematic review and meta-analysis

**DOI:** 10.3389/fendo.2024.1353334

**Published:** 2024-03-21

**Authors:** Botian Chen, Yunli Chen, Yuyang Wang, Qinghua Xin, Defu Ma

**Affiliations:** ^1^ School of Public Health, Peking University Health Science Center, Beijing, China; ^2^ School of Population Medicine and Public Health, Chinese Academy of Medical Sciences & Peking Union Medical College, Beijing, China; ^3^ Shandong Academy of Occupational Health and Occupational Medicine, Shandong First Medical University & Shandong Academy of Medical Sciences, Shandong, China

**Keywords:** rapid growth, triglyceride, total cholesterol, low density lipoprotein, high density lipoprotein

## Abstract

**Background & aims:**

Metabolic disease prevalence has increased in many regions, and is closely associated with dyslipidemia. Rapid growth refers to a significant increase in growth velocity above the normal range, particularly in infants and children, and is highly prevalent in congenital deficiency infants. But the association between dyslipidemia and rapid growth remains controversial. We performed this meta-analysis to investigate the lipid profile in subjects with and without postnatal rapid growth, and to determine what are the confounding factors.

**Methods:**

Medline, EMBASE, China National Knowledge Infrastructure Chinese citation database and WANFANG database were searched (last search in May 2021). Publication bias was examined by constructing funnel plots, Egger’s linear regression test and Begg’s rank correlation test.

**Results:**

The fixed effects model would be adopted if I^2^ is less than 25%, otherwise random effects model would be used. There were 11 articles involved with a total of 1148 participants (539 boys and 609 girls, mean age=7.4 years). Pooled analysis found that rapid growth was negatively associated with high-density lipoprotein cholesterol (HDL-C) (weighted mean difference=-0.068, 95%CI [-0.117, -0.020]), but not associated with triglycerides (TG), total cholesterol (TC), or low-density lipoprotein cholesterol (LDL-C). Stratified analysis suggested that increased TG were found in rapid growth subjects from developing countries. Higher TC was observed for rapid growth participants of follow-up age ≤8 years old, rapid growth duration ≤2 years, preterm, low birth weight, and from developing countries. But decreased TC was observed in small for gestational age (SGA) rapid growth subjects. Decreased LDL-C had been documented in rapid growth subjects of follow-up age >8 years old, from developed countries, and SGA. At last, rapid growth groups had lower HDL-C in infants of rapid growth duration >2 years and from developed countries.

**Conclusion:**

Rapid growth is associated with lipid profiles, particularly during early childhood, and this relationship is influenced by factors such as the duration of growth, the level of national development, and birth weight. These findings are significant for the development of strategies to prevent metabolic diseases.

This review was registered in PROSPERO International Prospective Register of Systematic Reviews (www.crd.york.ac.uk/prospero/) with the registration number CRD42020154240.

## Introduction

“Developmental plasticity” (1) describes the remarkable ability of organ development to adapt to environmental signals during sensitive periods, a phenomenon that allows for significant growth adjustments. Research indicates that certain infants, particularly those born preterm, with low birth weight (LBW), or small for gestational age (SGA), often experience a more rapid postnatal growth rate, termed “catch-up growth,” to align with their peers (2, 3). This accelerated growth is especially pronounced when complemented by adequate postnatal nutrition (4), serving as a common compensatory mechanism for these infants (5).

Despite the widespread recognition of rapid growth, there remains no universally accepted standard or definition for this term (6). Generally, any growth rate exceeding the normal growth velocity is classified as rapid growth. This growth can be measured by changes in body height or weight (in centimeters or kilograms). However, a more widely accepted method involves using Z-scores, which compare an individual’s growth to sex and age-specific norms in the general population. A weight or height gain exceeding 0.67 standard deviation scores (SDS) within the first 24 months of life, equivalent to a significant percentile shift (e.g., from the 2nd to the 9th, or 9th to the 25th percentile), is commonly indicative of rapid growth in current research (5). Additionally, accelerated growth in head circumference and body mass index (BMI) is also considered. While rapid growth typically occurs before the age of two, the duration of this growth phase varies significantly across studies.

There is a growing body of evidence suggesting that postnatal growth patterns can have a lasting impact on the programming of fetal gene expression, potentially leading to long-term metabolic risks (7) such as obesity (8–10), glucose metabolism disorders (11, 12), and cardiovascular disease (13). Moreover, substantial evidence indicates a link between these conditions and the dysregulation of cholesterol metabolism (14–18). However, the relationship between rapid postnatal growth and subsequent serum lipid levels remains a subject of debate.

Triglycerides (TG), a form of fat circulating in the bloodstream, serve as an energy source for the body’s cells. Elevated TG levels are associated with an increased risk of cardiovascular diseases (19). Total cholesterol (TC) is a comprehensive measure of cholesterol in the blood, including both low-density lipoprotein cholesterol (LDL-C) and high-density lipoprotein cholesterol (HDL-C). High TC levels are associated with atherosclerosis, a condition characterized by arterial plaque buildup that can lead to heart disease (20). LDL-C, often dubbed “bad cholesterol,” transports cholesterol to tissues and contributes to plaque formation (21), while HDL-C, known as “good cholesterol,” helps clear excess cholesterol from the bloodstream, thereby reducing the risk of heart disease (22). These lipid parameters were chosen for our study as they are pivotal indicators of cardiovascular health and are influenced by growth patterns, particularly during the rapid growth phase in early childhood. Elucidating the relationship between these lipid profiles and rapid growth is crucial for identifying potential risk factors and devising targeted interventions to foster cardiovascular health in children.

This meta-analysis aims to explore the lipid profiles of individuals with and without rapid postnatal growth and to identify influencing factors through subgroup analysis. The study parameters include TG, TC, LDL-C, and HDL-C in plasma.

## Methods

This review was registered in PROSPERO International Prospective Register of Systematic Reviews (www.crd.york.ac.uk/prospero/) with the registration number CRD42020154240.

We followed the Meta-analysis of Observational Studies in Epidemiology (MOOSE) guidelines for the conduct of systematic reviews and meta-analyses of observational studies (23).

### Eligibility criteria

Eligibility for inclusion in our study was based on the following criteria:

(1) Studies must compare the levels of TG, TC, LDL-C, and HDL-C between subjects with rapid growth and those without.
(2) The definition of rapid growth must be explicit, encompassing the duration of rapid growth (e.g., rapid growth in height, weight, and BMI, often termed “catch-up growth,” “catch-up weight gain,” and “catch-up BMI gain” respectively), and the extent of rapid growth (measured by standard deviation scores, SDS). For instance, in our study, a height gain of more than 0.67 SDS from birth to 2 years old was considered clinically significant rapid growth, with TG levels assessed at the age of 8 years. Here, “catch-up growth” refers to the rapid growth mode, SDS>0.67 indicates the extent of rapid growth, and the duration is 2 years (from birth to 2 years old). The follow-up age is set at 8 years, with a follow-up duration of 6 years.
(3) The studies must provide data on the number of subjects, means, standard deviations, and 95% confidence intervals [CIs] for TG, TC, LDL-C, or HDL-C in both the rapid growth and control groups.
(4) Only observational studies were considered for inclusion in the analysis.

### Search strategy and study selection

We systematically searched Medline (1966–May 2021) and Embase (1980–May 2021) for English studies (the start of their coverage). China National Knowledge Infrastructure Chinese citation database (CNKI) and WANFANG database were especially used to search articles in Chinese.

Considering that there are no universally accepted definitions of rapid growth, we reduced the restrictions of search terms and only entered “catch up growth” OR “rapid growth” OR “rapid weight gain” OR “rapid height gain”.

The following MeSH terms, words and phrases were used in the construction of the systematic search: (“catch-up growth” OR “rapid growth” OR “weight gain” OR “weight change” OR “height change” OR “height gain” OR “linear growth”) AND (“cholesterol” OR “lipoprotein” OR “triglyceride” OR “metabolic”) AND Humans [Mesh].

Two reviewers (Botian Chen and Defu Ma) independently screened titles and abstracts based on the inclusion criteria (The reviewers were trained to recognize the key words and concepts relevant to our research question, which included terms related to rapid growth, lipid profiles, and the specific age groups of interest. During the screening process, the reviewers manually excluded records that did not meet the criteria, such as those that were not original research studies, did not report the required lipid parameters, or were not focused on the relevant age groups. This manual screening was necessary to ensure that only studies with the highest relevance to our research question were included in the subsequent stages of the review.). We also searched the reference lists of all relevant articles to identify other potential studies. Then full-text articles were screened for eligibility. If necessary, we also emailed the authors when the required data were not reported in the articles.

All discrepancies were resolved by discussion and where needed, the third person (Qinghua Xin) arbitrated.

### Data extraction and quality assessment

A structured checklist was meticulously designed to independently capture pertinent data by two reviewers, Botian Chen and Defu Ma. Discrepancies in data extraction were addressed through collaborative dialogue. In cases where data were replicated across multiple studies, the study with the largest sample size or the most comprehensive data presentation was prioritized for inclusion. The extracted data encompassed:

1) Author and publication year.
2) Study population: sample size, country, preterm/term or not, SGA/AGA or not, LBW or not.
3) Rapid growth metrics: encompassing growth velocity in anthropometric parameters such as height, weight, and head circumference, the duration of rapid growth, and the extent of growth acceleration.
4) Outcome measures: Means and standard deviations or 95% CIs of TC, TG, LDL-C, and HDL-C in rapid growth groups and control groups, and the follow-up age.
5) Study design: Given the nature of rapid growth, only observational studies were considered. In cohort studies, the follow-up duration was determined from the end of the rapid growth period to the time of outcome assessment. Studies were classified as cross-sectional if the follow-up age coincided with the end of the rapid growth period (0-year follow-up duration).

The methodological quality of the studies was assessed using the Newcastle-Ottawa Scale (24). A total score of 9 stars was allocated, with studies scoring 7 or above deemed to have high methodological quality (Grade A), and those scoring 1 to 6 as low quality (Grade B). Studies with a follow-up duration exceeding three years were deemed sufficiently long-term, and additional stars were awarded to studies with less than 25% missed follow-ups.

### Statistical methods

Q statistics were used to assess the heterogeneity among the pooled studies, with P<0.10 indicating statistical significance. Additionally, the I^2^ index was calculated to assess the statistical heterogeneity across studies, representing the proportion of total variability attributable to true heterogeneity. The fixed effects model was chosen when the I^2^ value was less than 25%; otherwise, the random effects model was used.

We created forest plots to display the means and 95% CIs for the summary analysis. As previously determined, subgroup meta-analyses were conducted to examine potential effect moderators, including rapid growth duration, growth pattern, follow-up age, nationality, and subject characteristics. Weighted mean differences (WMDs) and associated 95% CIs were reported. Furthermore, meta-regression analysis was conducted to assess whether the effect of rapid growth on serum lipid level was related to rapid growth duration and follow-up age. Publication bias was assessed using funnel plots, Egger’s linear regression test, and Begg’s rank correlation test.

All analyses were conducted using STATA (version 11; Stata Corp, College Station, TX, USA), with P < 0.05 considered statistically significant unless otherwise specified.

## Results

### Description of studies

**Figure 1:**
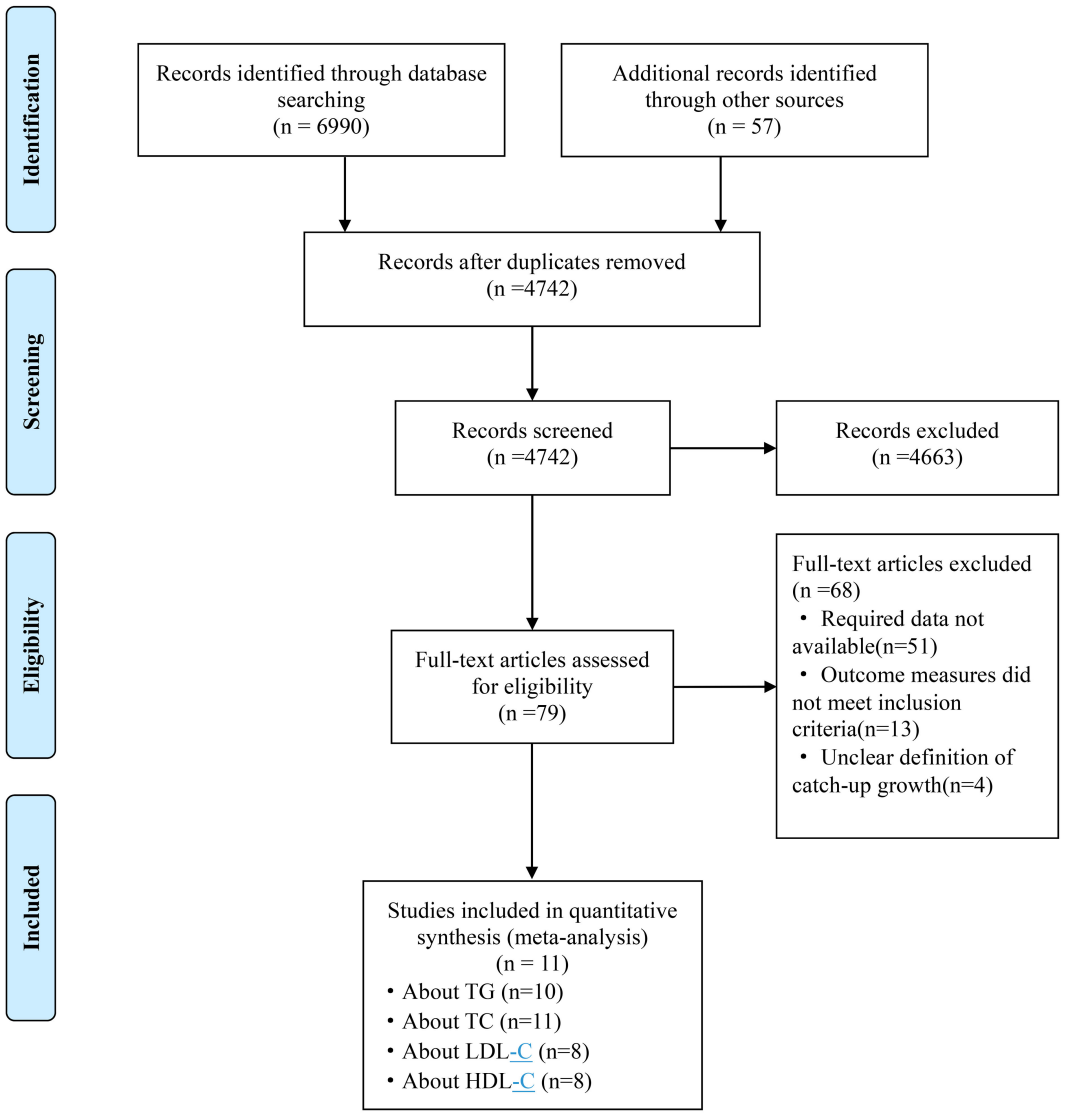
Flow chart for study selection. TG, triglyceride; TC, total cholesterol; LDL-C, low density lipoprotein cholesterol; HDL-C, high density lipoprotein cholesterol.

Initially, we identified 7047 studies, of which 2305 were excluded due to duplication. Upon further scrutiny, 11 studies were found to provide evidence of a relationship between rapid growth and lipid metabolism, comprising 9 English studies (25–33) and 2 Chinese studies (34, 35). Among these, 10 studies examined the associations between rapid growth and TG, 11 studies focused on TC, and 8 studies investigated LDL-C and HDL-C. The process of study selection is depicted in **Figure 1**.

### Basic features of included studies

Out of the 11 studies identified, seven were cross-sectional, three were cohort, and one was a case-control study. These studies were published between 2002 and 2017 and included a total of 1148 participants, with 1085 for TG, 1148 for TC, and 964 for LDL-C and HDL-C. The studies utilized population samples from various countries, including four from Italy, two from China, and one each from the Netherlands, England, Greece, Germany, and Cyprus. Sample sizes varied from 48 to 198 participants, with five studies having sample sizes under 100. Study design and exclusion and inclusion criteria were well-described in all included studies. And each of them gave a clear definition of rapid growth. The duration of rapid growth varied widely, ranging from 0.25 to 9.2 years, and the mean follow-up age also varied. Rapid growth ways included catch-up growth, catch-up weight gain, and catch-up BMI gain. The characteristics of the included studies are presented in **Table 1**.

**Table 1:** Main characteristics of the studies included in the Meta-analysis.

Source	Country	Sample size	Inclusion criteria	Follow-up age(year)	Rapid growth duration (year)	Rapid growth way	Rapid growth extent ^a^	Indicators
Cianfarani et al. (25), 2002	Italy	49	IUGR; term; SGA; no malformations or genetic disorders	9.2	0-9.2	height	SGA corrected height ≥0 z-score	TG/TC/LDL-C/HDL-C
Cianfarani et al. (26), 2003	Italy	135	SGA and AGA; no malformations, genetic disorders, family history of Type 2 diabetes, celiac disease, or hypothyroidism; karyotype was normal in all girls	8.6	0-8.6	height	SGA corrected height ≥0 z-score; short AGA (matched for sex, age [within 1 year], pubertal status, BMI [within 0.5 kg/m^2^], and height [within 0.25 z-score]) and non-rapid growth SGA served as controls	TG/TC/LDL-C/HDL-C
Toumba et al. (27), 2005	Cyprus	73	LBW (birth weight < 2500 g)	0-0.5/2	0-6	height	SGA had height SDS>-2; AGA served as controls	
Mohn et al. (28), 2007	Italy	48	Caucasian; singleton; term (gestation >37 weeks); SGA and AGA; no congenital anomalies (including Silver-Russel Syndrome), psychomotor delay, other chronic disorders, and/or autoimmune disease	4	0-4	height	SGA current length >10^th^ percentile; AGA and non-rapid growth SGA served as controls	TG/TC
Torre et al. (29), 2008	Italy	78	rapid growth SGA, and non-rapid growth SGA and AGA of the same gender and age (within 1 year); no malformations, genetic disorders, family history of Type 2 diabetes, celiac disease, or hypothyroidism; karyotype was normal in all girls	7.8	0-7.8	BMI	SGA with BMI=10^th^ to 75^th^ centile; short AGA and non-rapid growth SGA (matched gender, age [within 1 year], BMI [within 0.5 kg/m^2^] and pubertal stage) with BMI<10th centile served as controls	TG/TC/LDL-C/HDL-C
Gohlke et al. (30), 2009	Germany	63	preterm (gestation 23-31.3 weeks); VLBW (birth weight 350-990 g)	5.8	0-5.8	height	current height > -1 SDS, corrected for target height	TC
Leunissen et al. (31), 2010	Netherlands	198	Caucasian; singleton; term (gestation ≥36 weeks); SGA and AGA; no serious condition or had been receiving any treatment known to interfere with growth; no endocrine or metabolic disorders, chromosomal defects, syndromes or serious dysmorphic symptoms	20.9	0-9.2	height	SGA resulting in a normal adult height; AGA and non-rapid growth SGA served as controls	TG/TC/LDL-C/HDL-C
Wang et al. (34), 2015	China	126	preterm; SGA and AGA	0.25-1.5	0.25-1.5	weight	ΔSDS ≥1; 0.1≤ΔSDS <1 and ΔSDS <0.1 served as controls	TG/TC/LDL-C/HDL-C
Embleton et al. (32), 2016	Britain	102	preterm (gestation ≤34 weeks); no major neonatal morbidities (severe neurological abnormalities or lung disease at hospital discharge)	11.5	0-0.25	weight	ΔSDS>0.67; -0.67<ΔSDS≤0.67 and ΔSDS≤-0.67 served as controls	TG/TC/LDL-C/HDL-C
Wei et al. (35), 2016	China	160	Preterm; LBW; SGA and AGA	0.25-1.5	0.25-1.5	weight/height	ΔSDS≥1; 0.1≤ΔSDS <1 and ΔSDS <0.1 served as controls	TG/TC/LDL-C/HDL-C
Giapros et al. (33), 2017	Greece	116	SGA vs AGA with the same gestational age and gender	1	0-0.5	weight/height	ΔSDS >0.67; AGA served as controls	TG/TC/LDL-C/HDL-C

BMI, body mass index; SGA, small for gestational age; AGA, appropriate for gestational age; LBW, low birth weight; VLBW, very low birth weight; SDS, standard deviation scores; IUGR, intrauterine growth restriction; TG, triglyceride; TC, total cholesterol; LDL, low density lipoprotein cholesterol; HDL, high density lipoprotein cholesterol.

a Definitions of rapid groups and control groups in our meta-analysis might be different from original studies.

The quality of the included studies was assessed and is presented in **Supplementary Table 1**. All studies had more than 7 scores, belonging to high-quality research. The mean score was 8.64. Because the lipid profile was shown at the start of the studies, all the included three cohort studies were not assigned a score according to the criteria of the Newcastle-Ottawa scale. Moreover, one study was not assigned a score because it did not have adequate follow-up time.

### Meta-analysis of primary outcomes for the association between rapid growth and TG

**Figure 2:**
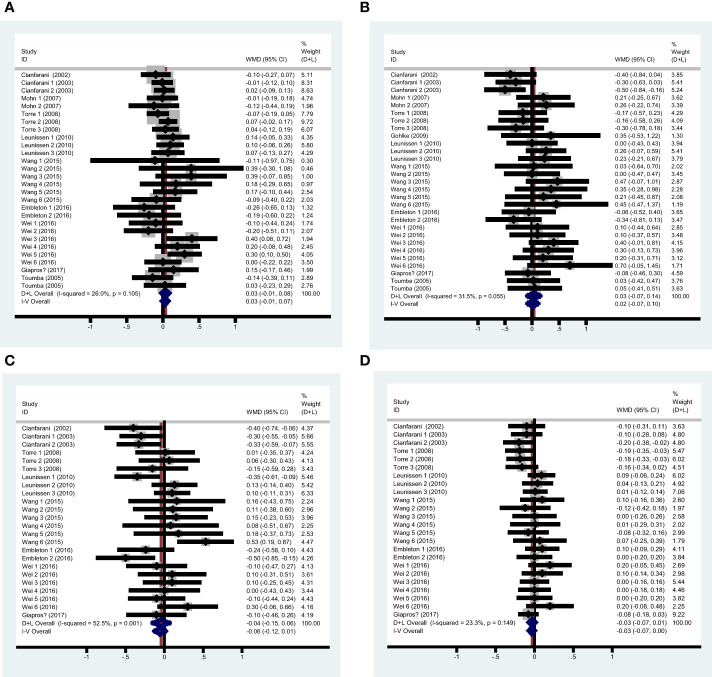
Pooled analysis forest diagram of the effect of rapid growth on TG/TC/LDL-C/HDL-C **(A)** Pooled analysis forest diagram of the effect of rapid growth on TG; **(B)** Pooled analysis forest diagram of the effect of rapid growth on TC; **(C)** Pooled analysis forest diagram of the effect of rapid growth on LDL-C; **(D)** Pooled analysis forest diagram of the effect of rapid growth on HDL-C.

Twenty-eight valid data were extracted from the 10 studies. **Table 2** shows the results of the summary analysis and stratified analysis. In the summary analysis, the heterogeneity test indicated a P-value of 0.105 and an I^2^ statistic of 26.0%, leading to the adoption of the random effects model. The results indicate that rapid growth was not significantly associated with TG, as shown by a pooled WMD of 0.034 (95%CI [-0.014, 0.081]). The forest diagram for the summary analysis is presented in **Figure 2A**. Furthermore, sensitivity analysis indicated that no single study significantly altered the pooled results.

**Table 2:** Pooled analysis and stratified analyses results of the effect of rapid growth on TG.

	Source		Heterogeneity			Summary/stratified analysis		
	df	Reference	Q	P	I^2^	WMD (95%CI)	Z	P
***Pooled analysis***	27	(25–29, 31–35)	36.49	0.105	26.0%	0.034 (-0.014, 0.081)	1.40	0.162
Follow-up age								
≤8 years old	19	(27–29, 33–35)	28.16	0.080	32.5%	0.050 (-0.017, 0.116)	1.46	0.144
>8 years old	7	(25, 26, 31, 32)	7.77	0.354	9.9%	0.015 (-0.043, 0.072)	0.49	0.621
Rapid growth duration								
≤2 years old	16	(27, 32–35)	26.14	0.052	38.8%	0.063 (-0.037, 0.164)	1.23	0.218
>2 years old	10	(25, 26, 28, 29, 31)	9.00	0.532	0.0%	0.019 (-0.024, 0.061)	0.87	0.385
Rapid growth way								
Weight	7	(32, 34)	8.68	0.277	19.3%	0.039 (-0.104, 0.181)	0.53	0.596
Height	10	(25–29, 31)	7.29	0.697	0.0%	0.014 (-0.036, 0.063)	0.53	0.594
Weight/height	6	(33, 35)	13.23	0.040	54.7%	0.115 (-0.040, 0.270)	1.45	0.146
BMI	1	(29)	0.072	0.072	69.0%	0.006 (-0.132, 0.144)	0.08	0.935
Term or not								
Preterm	13	(25, 29, 31)	22.79	0.044	43.0%	0.080 (-0.041, 0.200)	1.30	0.193
Term	5	(32, 34, 35)	5.43	0.366	7.9%	0.029 (-0.048, 0.106)	0.73	0.463
Undefined	7	(26, 27, 29, 33)	5.65	0.582	0.0%	0.012 (-0.036, 0.061)	0.50	0.618
LBW or not								
LBW	7	(27, 35)	17.02	0.017	58.9%	0.066 (-0.081, 0.212)	0.88	0.380
Undefined	19	(25, 26, 28, 29, 31–34)	18.36	0.499	0.0%	0.023 (-0.018, 0.063)	1.09	0.274
Rapid growth group (SGA or not)								
SGA	13	(25–29, 31, 33)	11.20	0.594	0.0%	0.017 (-0.024, 0.058)	0.81	0.417
Undefined	13	(32, 34, 35)	22.79	0.044	43.0%	0.080 (-0.041, 0.200)	1.30	0.193
Control group(SGA or AGA)(compared to SGA rapid growth group)								
SGA	5	(25, 26, 28, 29, 31)	4.63	0.462	0.0%	-0.013 (-0.073, 0.046)	-0.44	0.660
AGA	7	(26–29, 31, 33)	4.68	0.699	0.0%	0.044 (-0.012, 0.101)	1.53	0.125
Developed level								
Developed country	15	(25–29, 31–33)	14.03	0.524	0.0%	0.012 (-0.029, 0.053)	0.58	0.562
Developing country	11	(34, 35)	17.35	0.098	36.6%	0.122 (0.002, 0.242)	1.99	0.046

TG, triglyceride; df, degree of freedom; BMI, body mass index; SGA, small for gestational age; AGA, appropriate for gestational age; LBW, low birth weight.

Categorizing the data by follow-up age, definitions of rapid growth, and participant characteristics, the stratified analyses revealed that none of these factors—follow-up age, rapid growth duration, rapid growth method, term or preterm birth status, SGA/AGA status, or LBW status—significantly affected the difference in TG levels between the rapid growth group and the control group. However, when subjects were categorized by their countries as developed or developing, rapid growth was associated with elevated TG levels in developing countries (WMD 0.122, 95%CI [0.002, 0.242]). The linear regression analysis showed that follow-up age (r=-0.001, P=0.886) and rapid growth duration (r=0.002, P=0.641) were not significantly associated with TG.

To evaluate publication bias, a funnel plot was constructed and is presented in **Supplementary Figure 1A**. The results indicate a symmetrical distribution. Meanwhile, the Begg’s test and Egger’s test also didn’t find publication bias, and the P values of the two tests were 0.707 and 0.664, respectively.

### Meta-analysis of primary outcomes for the association between rapid growth and TC

Twenty-nine valid data were extracted from the 11 studies. The heterogeneity was assessed with an I^2^ value of 31.5%, leading to the use of the random effects model for the summary analysis. The summary analysis indicated that rapid growth was not significantly associated with total cholesterol (TC), with a WMD of 0.058 (95% CI: [-0.044, 0.161]). Sensitivity analysis indicated that the pooled results were not significantly altered by any individual study. **Table 3** shows the results of the summary analysis and stratified analyses, and **Figure 2B** shows the pooled analysis forest diagram.

**Table 3:** Pooled analysis and stratified analyses results of the effect of rapid growth on TC.

	Source		Heterogeneity			Summary/stratified analysis		
	df	Reference	Q	P	I^2^	WMD (95%CI)	Z	P
***Pooled analysis***	28	(25–35)	40.86	0.055	31.5%	0.032 (-0.074, 0.138)	0.59	0.554
Follow-up age								
≤8 years old	20	(27–30, 32–35)	16.63	0.677	0.0%	0.113 (0.004, 0.222)	2.03	0.042
>8 years old	7	(25, 26, 31, 32)	16.17	0.024	56.7%	-0.140(-0.354, 0.073)	-1.29	0.198
Rapid growth duration								
≤2 years old	16	(27, 32–35)	13.39	0.644	0.0%	0.126 (0.004, 0.248)	2.02	0.044
>2 years old	11	(25, 26, 28–31)	21.32	0.030	48.4%	-0.076 (-0.246, 0.095)	-0.87	0.386
Rapid growth way								
Weight	7	(32, 34)	7.00	0.428	0.1%	0.070 (-0.128, 0.269)	0.69	0.489
Height	11	(25–32)	21.43	0.029	48.7%	-0.041 (-0.215, 0.134)	-0.46	0.648
Weight/height	6	(33, 35)	5.18	0.521	0.0%	0.197 (0.020, 0.375)	2.18	0.030
BMI	1	(29)	0.00	0.972	0.0%	-0.166 (-0.456, 0.124)	-1.12	0.263
Term or not								
Preterm	14	(30, 32, 34, 35)	11.77	0.625	0.0%	0.175 (0.036, 0.315)	2.46	0.014
Term	5	(25, 28, 31, 32)	7.10	0.213	29.6%	0.101 (-0.106, 0.307)	0.96	0.339
Undefined	7	(26, 27, 29, 33)	6.12	0.525	0.0%	-0.207 (-0.348, -0.067)	-2.90	0.004
LBW or not								
LBW	8	(27, 30, 35)	4.22	0.837	0.0%	0.212 (0.045, 0.379)	2.49	0.013
Undefined	19	(25, 26, 28, 29, 31–34)	29.39	0.060	35.4%	-0.038 (-0.165, 0.089)	-0.59	0.556
Rapid growth group (SGA or not)								
SGA	13	(25–29, 31–33)	20.97	0.074	38.0%	-0.074 (-0.213, 0.065)	-1.04	0.297
Undefined	14	(30, 32, 34, 35)	11.77	0.625	0.0%	0.175 (0.036, 0.315)	2.46	0.014
Control group(SGA or AGA)(compared to SGA rapid growth group)								
SGA	5	(25, 26, 28, 29, 31)	5.18	0.394	3.5%	-0.177 (-0.346, -0.008)	-2.01	0.045
AGA	7	(26–29, 31–33)	13.69	0.057	48.9%	-0.002 (-0.203, 0.198)	-0.02	0.982
Developed level								
Developed country	16	(25–33)	23.04	0.113	30.5%	-0.080 (-0.205, 0.044)	-1.26	0.207
Developing country	11	(34, 35)	5.08	0.927	0.0%	0.254 (0.097, 0.410)	3.18	0.001

TC, total cholesterol; df, degree of freedom; BMI, body mass index; SGA, small for gestational age; AGA, appropriate for gestational age; LBW, low birth weight.

Stratified analyses revealed a significant association between rapid growth and TC for participants with a follow-up age less than 8 years (WMD 0.113, 95% CI: [0.004, 0.222]) and for those with a rapid growth duration of less than 2 years (WMD 0.126, 95% CI: [0.004, 0.248]). Additionally, subjects who were preterm (WMD 0.175, 95% CI: [0.036, 0.315]), had LBW (WMD 0.212, 95% CI: [0.045, 0.379]), or were from developing countries (WMD 0.254, 95% CI: [0.097, 0.410]) showed higher TC levels in the rapid growth groups. However, infants with SGA who experienced rapid growth had lower TC levels compared to those without rapid growth (WMD -0.177, 95% CI: [-0.346, -0.008]). However, the different methods of rapid growth did not appear to influence TC levels. Meta-regression analyses showed no significant association between TC levels and follow-up age (r=-0.010, P=0.257) or rapid growth duration (r=-0.005, P=0.538).

The funnel plot is presented in **Supplementary Figure 1B**, with P-values for the Begg’s test and Egger’s test being 0.034 and 0.011, respectively. To address potential publication bias, the trim and fill method was used to recalculate the summary analysis, yielding results indicating no association between rapid growth and TC (WMD -0.062, 95% CI: [-0.172, 0.048]), consistent with the initial findings (WMD 0.058, 95% CI: [-0.044, 0.161]).

### Meta-analysis of primary outcomes for the association between rapid growth and LDL-C

Twenty-four valid data were recruited from the 8 studies. The heterogeneity test indicated a P-value less than 0.1 (I^2 ^= 52.5%), leading to the adoption of the random effects model for the combined analysis. The summary analysis showed no significant influence of rapid growth on LDL-C (WMD -0.042, 95%CI [-0.147, 0.062]). Sensitivity analysis indicated that the pooled results were not significantly altered by any single study. **Table 4** shows the results of the summary analysis and stratified analyses, and **Figure 2C** shows the summary analysis forest diagram.

**Table 4:** Pooled analysis and stratified analyses results of the effect of rapid growth on LDL-C.

	Source		Heterogeneity			Summary/stratified analysis		
	df	Reference	Q	P	I^2^	WMD (95%CI)	Z	P
***Pooled analysis***	23	(25, 26, 29, 31–35)	48.42	0.001	52.5%	-0.042 (-0.147, 0.062)	-0.80	0.424
Follow-up age								
≤8 years old	15	(29, 32–35)	13.04	0.599	0.0%	0.084 (-0.016, 0.184)	1.65	0.099
>8 years old	7	(25, 26, 31, 32)	20.89	0.004	66.5%	-0.220 (-0.389, -0.050)	-2.54	0.011
Rapid growth duration								
≤2 years old	14	(32–35)	24.43	0.041	42.7%	0.033(-0.104, 0.170)	0.47	0.683
>2 years old	8	(25, 26, 29, 31)	19.24	0.014	58.4%	-0.136 (-0.287, 0.014)	-1.77	0.076
Rapid growth way								
Weight	7	(32, 34)	20.45	0.005	65.8%	0.047 (-0.215, 0.309)	0.35	0.724
Height	6	(25, 26, 29, 31)	17.39	0.008	65.5%	-0.177 (-0.355, 0.001)	-1.95	0.051
Weight/Height	6	(33, 35)	3.99	0.679	0.0%	0.025 (-0.115, 0.165)	0.35	0.727
BMI	1	(29)	0.04	0.842	0.0%	0.035 (-0.219, 0.290)	0.27	0.786
Term or not								
Preterm	13	(32, 34, 35)	23.88	0.032	45.6%	0.045 (-0.102, 0.191)	0.60	0.552
Term	3	(25, 31)	12.63	0.006	76.2%	-0.114 (-0.386, 0.157)	-0.83	0.409
Undefined	5	(26, 29, 33)	5.15	0.397	3.0%	-0.182 (-0.312, -0.053)	-2.68	0.007
LBW or not								
LBW	5	(35)	3.40	0.638	0.0%	0.048 (-0.104, 0.200)	0.62	0.536
Undefined	17	(25, 26, 29, 31–34)	42.78	0.001	60.3%	-0.069 (-0.198, 0.060)	-1.05	0.292
Rapid growth group (SGA or not)								
SGA	9	(25, 26, 29, 31–33)	19.25	0.023	53.2%	-0.133 (-0.271, 0.004)	-1.90	0.057
Undefined	13	(32, 34, 35)	23.88	0.032	45.6%	0.045 (-0.102, 0.191)	0.60	0.552
Control group(SGA or AGA)(compared to SGA rapid growth group)								
SGA	4	(25, 26, 29, 31)	3.60	0.462	0.0%	-0.268 (-0.407, -0.129)	-3.79	<0.001
AGA	4	(26, 29, 31–33)	8.49	0.075	52.9%	-0.025 (-0.209, 0.158)	-0.27	0.786
Developed level								
Developed country	11	(25, 26, 29, 31–33)	23.53	0.015	53.3%	-0.168 (-0.297, -0.04)	-2.56	0.010
Developing country	11	(34, 35)	10.00	0.530	0.0%	0.132 (0.014, 0.250)	2.20	0.028

LDL-C, low density lipoprotein cholesterol; df, degree of freedom; BMI, body mass index; SGA, small for gestational age; AGA, appropriate for gestational age; LBW, low birth weight.

Stratified analyses revealed that LDL-C were lower in subjects with a follow-up age greater than 8 years (WMD -0.220, 95% CI: [-0.389, -0.050]), in those with small for gestational age (SGA) who experienced rapid growth compared to those without (WMD -0.268, 95% CI: [-0.407, -0.129]), and in participants from developed countries (WMD -0.168, 95% CI: [-0.297, -0.04]). In contrast, participants from developing countries with rapid growth exhibited higher LDL-C (WMD 0.132, 95%CI [0.014, 0.250]). Meta-regression analyses showed no significant association between LDL-C and follow-up age (r=-0.011, P=0.136) or rapid growth duration (r=-0.005, P=0.490).

The funnel plot, presented in **Supplementary Figure 1C**, indicates a symmetrical distribution. Additionally, the Begg’s test (P=0.333) and Egger’s test (P=0.307) did not detect any publication bias.

### Meta-analysis of primary outcomes for the association between rapid growth and HDL-C

The studies included for HDL-C analysis were the same as those for LDL-C. In the summary analysis, the heterogeneity test indicated a P-value of 0.149 and an I^2^ statistic of 23.3%, leading to the use of the random effects model. The results indicated no significant difference in HDL-C between the rapid growth and control groups in the summary analysis (WMD -0.030, 95%CI [-0.074, 0.015]). The Begg’s test yielded a P-value of 0.206, while the funnel plot and Egger’s test (P=0.098) indicated the presence of publication bias. To address the publication bias, the trim and fill method was applied, revealing a negative association between rapid growth and HDL-C (WMD -0.068, 95%CI [-0.117, -0.020]). Sensitivity analysis indicated that no single study significantly influenced the pooled results. **Table 5** presents the results of both the summary and stratified analyses, while **Figure 2D** illustrates the forest diagram from the summary analysis. The funnel plot is displayed in **Supplementary Figures 1D, E**.

**Table 5:** Pooled analysis and stratified analyses results of the effect of rapid growth on HDL-C.

	Source		Heterogeneity			Summary/stratified analysis		
	df	Reference	Q	P	I^2^	WMD (95%CI)	Z	P
***Pooled analysis***	23	(25, 26, 29, 31–35)	30.00	0.149	23.3%	-0.030 (-0.074, 0.015)	-1.78	0.075
*Trim and fill method*	29	–	52.609	0.005	–	-0.068 (-0.117, -0.020)	-2.755	0.006
Follow-up age								
≤8 years old	15	(32–35)	19.56	0.190	23.3%	-0.048 (-0.097, 0.000)	-1.95	0.051
>8 years old	7	(25, 26, 29, 31, 32)	9.62	0.211	27.2%	-0.015 (-0.086, 0.057)	0.41	0.682
Rapid growth duration								
≤2 years old	14	(32–35)	10.05	0.758	0.0%	0.007 (-0.045, 0.060)	0.27	0.788
>2 years old	8	(25, 26, 29, 31)	14.97	0.060	46.6%	-0.084 (-0.159, -0.009)	-2.18	0.029
Rapid growth way								
Weight	7	(32, 34)	2.72	0.910	0.0%	0.017 (-0.070, 0.104)	0.38	0.704
Height	6	(25, 26, 29, 31)	9.92	0.128	39.5%	-0.050 (-0.132, 0.032)	-1.20	0.229
Weight/height	6	(33, 35)	7.26	0.297	17.4%	0.014 (-0.062, 0.090)	0.05	0.960
BMI	1	(29)	0.00	0.950	0.0%	-0.187 (-0.296, -0.078)	-3.36	0.001
Term or not								
Preterm	13	(32, 34, 35)	6.62	0.921	0.0%	0.036 (-0.025, 0.097)	1.16	0.244
Term	3	(25, 31)	2.17	0.537	0.0%	0.023 (-0.056, 0.103)	0.57	0.567
Undefined	5	(26, 29, 33)	2.78	0.734	0.0%	-0.138 (-0.199, -0.078)	-4.46	<0.001
LBW or not								
LBW	5	(35)	3.54	0.617	0.0%	0.054 (-0.030, 0.139)	1.25	0.210
Undefined	17	(25, 26, 29, 31–34)	21.23	0.216	19.9%	-0.056 (-0.098, -0.014)	-2.20	0.028
Rapid growth group (SGA or not)								
SGA	9	(25, 26, 29, 31–33)	14.97	0.092	39.9%	-0.082 (-0.146, -0.018)	-2.53	0.012
Undefined	13	(32, 34, 35)	6.62	0.921	0.0%	0.036 (-0.025, 0.097)	1.16	0.244
Control group(SGA or AGA)(compared to SGA rapid growth group)								
SGA	4	(25, 26, 29, 31)	7.65	0.105	47.7%	-0.087 (-0.194, 0.020)	-1.59	0.112
AGA	4	(26, 29, 31, 33)	7.31	0.120	45.3%	-0.079 (-0.166, 0.007)	-1.80	0.072
Developed level								
Developed country	11	(25, 26, 29, 31–33)	18.46	0.071	40.4%	-0.065 (-0.125, -0.004)	-2.10	0.036
Developing country	11	(34, 35)	6.07	0.869	0.0%	0.032 (-0.035, 0.100)	0.94	0.347

HDL-C, high density lipoprotein cholesterol; df, degree of freedom; BMI, body mass index; SGA, small for gestational age; AGA, appropriate for gestational age; LBW, low birth weight.

Subgroup analyses identified that a rapid growth duration exceeding 2 years (WMD -0.084, 95% CI: [-0.159, -0.009]) and participants from developed countries (WMD -0.065, 95% CI: [-0.125, -0.004]) were associated with lower HDL-C in the rapid growth groups. Furthermore, rapid growth in SGA infants was associated with lower HDL-C compared to control groups (WMD -0.082, 95% CI: [-0.146, -0.018]). However, when the control groups were further refined, no significant difference in HDL-C was observed between rapid growth SGA infants and those with no rapid growth (SGA or AGA). Linear regression analysis revealed no significant association between HDL-C and follow-up age (r=0.001, P=0.787) or rapid growth duration (r=0.000, P=0.881).

## Discussion

This is the first meta-analysis to investigate the association between rapid growth and subsequent serum lipid levels. Our pooled analysis findings suggested that rapid growth was associated with lower HDL-C. Subgroup analyses revealed that follow-up age, definitions of rapid growth, and participant characteristics influence the associations between rapid growth and TG, TC, LDL-C, and HDL-C.

Dyslipidemia is an established risk factor for many noncommunicable diseases. In clinical research, LDL cholesterol is often calculated using the Friedewald formula: LDL cholesterol (mmol/l) = total cholesterol – HDL cholesterol -0.45*triglycerides (36). Triglyceride is regarded as an energy source for peripheral tissues. They mobilize from adipose tissue in the fasting/starved state. The rise of triglyceride is a risk factor for type II diabetes mellitus (37), metabolic syndrome (38), cardiovascular (39–42), cognitive function (43), and so on. TC is also positively associated with coronary heart disease (CHD) (44) and stroke (45, 46). Furthermore, lipids are transported in the blood by lipoproteins, which include HDL-C and LDL-C. However, the correlation between HDL-C concentration and non HDL-C in vascular risk is opposite. Many therapies reduce the risk of CHD, ischemic heart disease and stroke by lower LDL-C levels (47, 48) and elevating HDL-C levels (49, 50). Dyslipidemia prevalence has increased in many regions (51), especially in developing countries (52, 53). Early childhood represents a potentially modifiable critical period. A thorough understanding of the association between rapid growth and lipid metabolism may lead to new preventive strategies to combat metabolic disease.

It remains uncertain whether there is a specific time window during which rapid growth is beneficial for children (5). Some researchers thought that rapid growth early after birth is a normal adaptation to restore body size and does not impact metabolism (5, 32, 54, 55). Another explanation suggests that prolonged rapid growth is due to sustained higher caloric intake, a known risk factor for metabolism disturbance (56, 57). Our research found that subjects who experienced rapid growth before the age of 2 had higher TC, while those with rapid growth for more than 2 years had lower HDL-C. However, it is important to note that growth is continuous process, and determining the rapid growth at precise time points will always be challenging. Children who experience rapid growth for more than 2 years may have already begun this growth pattern before the age of 2. Conversely, we found that follow-up age did not affect the correlation between rapid growth and TG or HDL-C. However, the rapid growth group exhibited higher TC in subjects under 8 years of age and lower LDL-C in those over 8 years. In conclusion, early-life rapid growth appears to have a long-term beneficial effect on lipid metabolism.

SGA is typically defined as birth weight and/or length at least 2 standard deviations (SDs) below the mean for gestational age or at the 10th percentile. SGA subjects are a heterogeneous population and growth pattern plays an important role in influencing the lipid metabolic risk. It appears that SGA children are prone to various metabolic aberrations. Cianfarani et al. (26) observed that SGA children without rapid growth showed significantly reduced levels of TC than short-AGA subjects. Rabinowicz et al (58) found SGA preterm infants showed higher TG levels compared with age-matched AGA infants. However, the results of our research revealed that there is no difference in lipid profile between rapid growth SGA subjects and no rapid growth AGA infants, but rapid growth SGA showed lower TC and LDL-C when compared to no rapid growth SGA, suggesting that rapid growth is relatively safe for SGA to catch up with their peers. Our findings have significant implications for the feeding practices of SGA infants.

Our study focused solely on the impact of rapid growth on lipid metabolism. To date, epidemiological and clinical studies have shown that many diseases may originate from developmental trajectories in early life (4, 5). For example, some studies have reported that catch-up weight gains predicted increased risk for high glucose concentrations, high blood pressure (59), and cardiovascular disease (60). It has been suggested that interventions aimed at limiting excessive postnatal weight gain might prevent the development of central obesity, insulin resistance, and cardiovascular disease risks (61). However, other evidence showed that rapid growth may have positive effects on development and growth. A cohort study in southern Brazil presented that for SGA children, the rapid growth group had 65% fewer subsequent hospital admissions and 75% lower mortality to age 5 years (62). Rapid growth in very low birth weight infants may reduce bronchial responsiveness (63), decrease short stature risk, and enhance neurodevelopment outcomes (64). Moreover, rapid growth has a negative relationship with insulin resistance and a positive relationship with prealbumin and IGF-1 (65). In summary, various health outcomes can be linked to early-life growth trajectories, and the mechanisms that connect early growth patterns with later outcomes are intricate.

### Limitations

Our study had several limitations. The first limitation is the heterogeneity, as discussed above. Differences in follow-up age, definitions of rapid growth, characteristics of participants, and the chosen control groups, would all affect the interpretation of the conclusions. Although we employed a stratified analysis method, it may not have adequately accounted for all confounding factors. Efforts should be directed towards standardizing the definition of rapid growth, encompassing growth durations, anthropometric parameters, and the extent of growth, to enhance the comparability of results across studies. Secondly, the majority of the included studies have small sample sizes. A large-scale, population-based prospective cohort study is needed to illustrate relative problems. Thirdly, our study is based solely on observational studies, which may not account for all confounding factors, including genetic variation (66–68). Therefore, further exploration of causal inferences is warranted. What’s more, the rapid growth time in our study refers to the time to evaluate the rapid growth, and the specific rapid growth time can only be determined through cohort study. In addition, publication bias is potential limitation.

## Conclusions

In conclusion, our study demonstrates a significant association between rapid growth in early childhood and changes in lipid profiles, which may have profound implications for long-term cardiovascular health. These findings emphasize the importance of early growth patterns in the development of metabolic disorders and suggest that interventions targeting rapid growth could be crucial in reducing the risk of cardiovascular diseases in later life. Our research contributes to the growing body of evidence linking early life growth trajectories with adult health outcomes, offering a novel perspective on the critical window of early childhood for preventive strategies. Furthermore, our study provides actionable information for clinicians to monitor and manage the lipid profiles of children experiencing rapid growth, potentially leading to improved health outcomes and a reduction in the burden of cardiovascular diseases in the population.

## Author contributions

BC: Conceptualization, Data curation, Formal Analysis, Methodology, Visualization, Writing – original draft, Writing – review & editing. DM: Conceptualization, Data curation, Formal Analysis, Funding acquisition, Methodology, Writing – original draft, Writing – review & editing. YC: Data curation, Formal Analysis, Writing – review & editing. WY: Writing – review & editing. QX: Writing – review & editing.
