# Supplementary material for: The association between rapid growth and lipid profile: a systematic review and meta-analysis
Source: Front Endocrinol (Lausanne). 2024 Mar 21;15:1353334. doi: 10.3389/fendo.2024.1353334 (PMC10991823; doi:10.3389/fendo.2024.1353334)
Supplement: Supplementary file 1 [file Image_1.pdf]

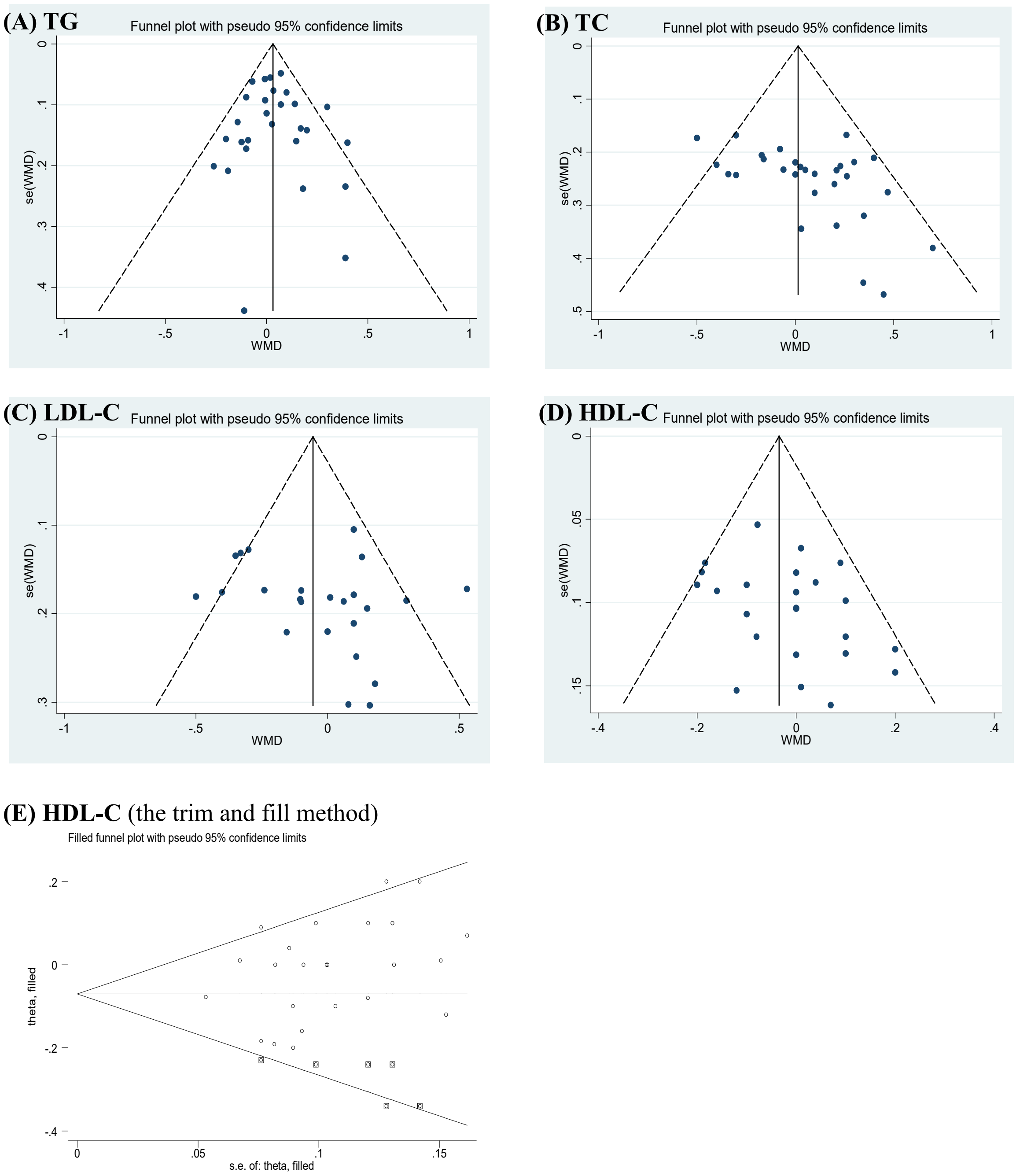

Supplementary Figure 1. Funnel plot of the effect of rapid growth on TG/TC/LDL-C/HDL-C

(A) Funnel plot of the effect of rapid growth on TG; (B) Funnel plot of the effect of rapid growth on TC; (C) Funnel plot of the effect of rapid growth on LDL-C; (D) Funnel plot of the effect of rapid growth on HDL-C; (E) Funnel plot of the effect of rapid growth on HDL-C by the trim and fill method.
