# Supplementary material for: The association between rapid growth and lipid profile: a systematic review and meta-analysis
Source: Front Endocrinol (Lausanne). 2024 Mar 21;15:1353334. doi: 10.3389/fendo.2024.1353334 (PMC10991823; doi:10.3389/fendo.2024.1353334)
Supplement: Supplementary file 2 [file Table_1.docx]

**Supplementary table 1 Quality Assessment of Included Studies Using the Newcastle-Ottawa Scale**

| **Author, year** | **Selection** | **Comparability** | **Outcome/Exposure** | **Total Score** |
| --- | --- | --- | --- | --- |
| Cianfarani et al, 2002 | **** | ** | *** | 9 |
| Cianfarani et al, 2003 | **** | ** | *** | 9 |
| Toumba et al, 2005 | *** | ** | ** | 8 |
| Mohn,2007 | **** | ** | *** | 9 |
| Torre et al, 2008 | **** | ** | *** | 9 |
| Gohlke et al, 2009 | **** | ** | *** | 9 |
| Leunissen et al,2010 | **** | ** | *** | 9 |
| Wang et al, 2015 | **** | ** | *** | 9 |
| Embleton et al,2016 | *** | ** | ** | 8 |
| Wei et al, 2016 | **** | ** | *** | 9 |
| Giapros et al, 2017 | *** | ** | ** | 7 |
